# Application of next-generation metagenomic sequencing in the diagnosis and treatment of acute spinal infections

**DOI:** 10.1016/j.heliyon.2023.e13951

**Published:** 2023-02-22

**Authors:** Chen Wang, Jinquan Hu, Yifei Gu, Xinwei Wang, Yu Chen, Wen Yuan

**Affiliations:** Department of Spinal Orthopedics, Shanghai Changzheng Hospital, Shanghai, China

**Keywords:** Spinal, Infectious, mNGS, Culture, Microbiology, Antibiotic

## Abstract

**Objectives:**

The purpose of this study was to verify the value of metagenomic next-generation sequencing (mNGS) in detecting the pathogens causing acute spinal infection by reviewing the results of mNGS in 114 patients.

**Methods:**

A total of 114 patients were included from our hospital. Samples (tissue/blood) were sent for mNGS detection, and the remaining samples were sent to the microbiology laboratory for pathogen culture, smear, histopathological analysis, and other tests. Patients' medical records were reviewed to determine their rates of detection, time needed, guidance for antibiotic treatment and clinical outcomes.

**Results:**

mNGS showed a satisfying diagnostic positive percent agreement of 84.91% (95% confidence interval (CI): 6.34%–96.7%), compared to 30.19% (95% CI: 21.85%–39.99%) for culture and 43.40% (95% CI: 31.39%–49.97%) for conventional methods (p < 0.0125), and mNGS was found positive in 46 culture and smear negative samples. The time required for pathogen identification using mNGS ranged from 29 h to 53 h, which showed an advantage over culture (90.88 ± 8.33 h; P < 0.05). mNGS also played an important role in optimizing antibiotic regimens in patients with negative results obtained using conventional methods. The treatment success rate (TSR) of patients using mNGS-guided antibiotic regimens (20/24, 83.33%) was significantly higher than that of patients using empirical antibiotics (13/23, 56.52%) (P < 0.0001).

**Conclusions:**

mNGS shows promising potential in the pathogenic diagnosis of acute spinal infections and may enable clinicians to make more timely and effective adjustments to antibiotic regimens.

## 1 Introduction

Acute spinal infection is a complex diagnosis with treatment challenges that usually require the multidisciplinary collaboration of a spinal surgeon, radiologist, and infectious disease specialist. The overall incidence of acute spinal infection is approximately 2.2/100,000 population per year [1,2]. Most patients with early-diagnosed acute spinal infections can be cured by conservative treatment with antibiotics and bed rest [3]. The pathogen must be diagnosed early to guide the application of effective antibiotics. However, the traditional culture method is time-consuming and has a low positive detection rate [4].

With advances in molecular biology, the value of metagenomic next-generation sequencing (mNGS) has attracted increasing attention, especially for the detection of complex, rare, atypical, or slow-growing microorganisms. Previously, mNGS was mainly used for the clinical evaluation of sterile body fluids, including cerebrospinal fluid, blood, and joint effusion fluid [[5], [6], [7]]. For nonsterile tissues, such as infected intervertebral discs or paravertebral abscesses, the application of mNGS is quite limited. In addition to human genetic interference and other common challenges, the interpretation of mNGS results (colonization vs. infection) remains controversial and uncertain. In recent years, the value of mNGS in the detection of pathogens and diagnosis of infectious diseases, such as respiratory tract infections and tuberculous meningitis, has been confirmed [8,9]. In a retrospective analysis of ninety-nine children with bacterial meningitis, mNGS technology detected ten different bacterial pathogens [10]. Furthermore, a large-scale prospective study conducted on specimens from 511 infected patients showed that mNGS is more sensitive in the detection of pathogens than culture, and mNGS is less affected by prior antibiotic exposure than culture [11]. However, evidence of the efficacy of mNGS in the detection of infectious pathogens in infected spinal tissue is still lacking.

In this study, we summarized the mNGS results from 114 patients. We interpreted the results of mNGS to investigate the value of mNGS in the diagnosis and treatment of acute spinal infections.

## 2 Materials and methods

### 2.1 Study design and participants

All patients suspected of having an acute (symptom duration <21 days) acute spinal infection who were treated at the Spinal Orthopaedics Department of Shanghai Changzheng Hospital were included from June 23, 2019, to February 23, 2021. After signing the informed consent form, tissue samples were obtained using computed tomography (CT)-guided percutaneous biopsy. The diagnostic criteria for acute spinal infection were as follows: 1. fever (rectal temperature >38.5 °C or axillary temperature >38.0 °C); 2. localized pain in the spine; 3. at least one of the following blood test results: leukocytes >100 cells/mm or leukocytes 10–100 cells/mm with an elevated erythrocyte sedimentation rate (ESR) (>20 mm/h) or C-reactive protein (CRP) level (>10 mg/L); 4. typical magnetic resonance imaging (MRI) findings of spondylodiscitis, including (1) hypointense vertebral bodies and discs with loss of endplate definition on T1-weighted images, (2) hyperintense vertebral bodies and discs with loss of endplate definition on T2-weighted images or short tau inversion recovery (STIR) images, and (3) contrast enhancement of the vertebral body and disc [12]; and 5. a positive pathogen test, including blood/tissue culture, smear examination, pathological analysis, or mNGS. A patient who met diagnostic criteria 1, 2, 3 and 4 at the same time was considered to have a clinical acute spinal infection. A patient with a clinical acute spinal infection who also met criterion five was considered to have a definite acute spinal infection. The final diagnosis and invasive procedures were performed by two senior orthopedic surgeons (Yu Chen and Xinwei Wang).

The extraction process was strictly performed using aseptic principles; the surgeon sterilized the patient's skin and wore sterile gloves. The mNGS process was also conducted in a sterile laboratory to minimize the possibility of contamination.

Despite the use of the aforementioned measures, the presence of the abovementioned microbial nucleic acids was detected in some samples due to epidermal colonization by bacteria and environmental microorganisms, and thus we analyzed blank samples simultaneously (blank samples are identical to the test sample material and operating environment, taken from the surrounding environment such as “healthy skin/air”). This method provided a reference for background microorganisms in the environment and allowed the filtering of results with low nucleic acid numbers and the detection of common background microorganisms by establishing a positive threshold for interpretation of the results.

Tissue samples stored sterile in a dry ice box were sent to a local pathogen detection laboratory (BGI-Shenzhen, China) for mNGS. The remaining tissue samples and blood samples were sent to the microbiology laboratory of our hospital for pathogen culture and smear examination. The surgeons ordered additional laboratory tests, such as the T-SPOT and other pathological tests, based on their clinical judgment of necessity. mNGS-positive specimens were verified by specific polymerase chain reaction (PCR) to exclude the possibility of false positives.

The baseline information of patients was extracted from the electronic medical record system; extracted data included age, sex, white blood cell count, ESR, CRP level, time of antibiotic use (days) before pathogen testing, and whether antibiotics were changed according to the etiological results and treatment results.

Written informed consent was obtained from the patients before participation in the study. This study was approved by the Ethical Review Committee of Changzheng Hospital.

### 2.2 DNA extraction, library preparation, and sequencing

Tissue samples were flash-frozen and stored at −20 °C before DNA extraction and library construction. Approximately 0.5 mg of the tissue sample was cut into small pieces and mixed with 0.7 mL of lysis buffer and 1 g of 0.5 mm glass beads in a 1.5-mL microcentrifuge tube. Then, the 1.5-mL microcentrifuge tube was attached to a horizontal platform on a VORTEX-GENIE 2 VORTEX MIXER 12 (Scientific Industries, USA) and agitated vigorously at 2800–3200 RPM for 30 min. Subsequently, 0.3 mL of lysate was placed into a new 1.5-mL microcentrifuge tube, and DNA was extracted using the TIANamp Micro DNA Kit (DP316, TIANGEN BIOTECH) according to the manufacturer's instructions. According to the manufacturer's instructions, DNA was extracted from 300 μL of infectious tissues using the TIANamp Micro DNA Kit (DP316, TIANGEN BIOTECH, Beijing, China). We processed potentially infectious tissues from patients with a suspected fungal infection with glass beads before DNA preparation (Z250465, SIGMA, St. Louis, MO, USA).

DNA libraries were constructed by fragmenting DNA into 100–150 bp fragments, which were flat-end repaired and barcode adapter ligated; these modified fragments were unbiasedly amplified with PCR using BGI reagents (BGI, Tianjin, China). The quality of the DNA libraries was analyzed with an Agilent 2100 instrument (Agilent Technologies, Santa Clara, CA, USA) and a Qubit 2.0 system. Qualified libraries (median sequence size ranging from 200 to 300 bp, >2.0 ng/μL) with different barcode labels were pooled. Sequencing was performed using the MGISEQ-2000 platform (MGI, Shenzhen, China).

Low-quality and short (length <35 bp) reads and human host sequences mapped to the human reference genome (hg19) using the Burrows–Wheeler Alignment tool (version 0.7.10-r789) were removed. After removing low-complexity reads, the remaining reads were classified by simultaneous alignment to a locally built reference database, the Microbial Genome Database. The reference database for microbial classification maintained by BGI (Wuhan, China) contains 1798 whole-genome sequences of viral taxa, 6350 bacterial genomes or scaffolds, 1064 genomes of fungi, and 234 genomes of parasites. Data analytics algorithms were used to exclude microorganisms that were not significantly related to the infection. Genus-/species-specific reads uniquely aligned to infection-related microorganisms were reported.

If the detected pathogen reached any of the following thresholds, it was determined to be an infectious pathogen: 1. the number of unique bacterial reads (species level) was more than 10 times higher than that of other bacteria [13]; 2. the number of unique viral reads was more than 10 times higher than that of other viruses, or at least 3 unique reads were detected; 3. The number of unique fungal reads (species level) was more than 5 times higher than that of other fungi [14,15]; 4. at least 1 unique read (genus level) belonging to the *Mycobacterium tuberculosis* complex (MBTC) was detected [16]. For some microorganisms that are not commonly observed clinically, we use the following methods to make a judgment: 1. Assess the relevant literature for reports of pathogenicity; 2. Ask for a consultation with an infection specialist to assist in the diagnosis; 3. Consult the microbiology department of our hospital regarding whether the identified microorganisms have been detected recently; if so, environmental contamination is possible, but if not, the likelihood that the organisms are pathogenic increases; 4. In cases of multiple infections observed in the mNGS results, such as Aspergillus and a virus, we perform diagnostic treatment and observe the patient's response to antiviral and antifungal treatment.

### 2.3 Statistical analysis

The Mann–Whitney *U* test and the chi-square test were used to analyze baseline characteristics. A 2 × 2 contingency table was derived based on the extracted data to determine the sensitivity, specificity, positive predictive value (PPV), and negative predictive value (NPV). Sensitivities were compared using McNemar's test. The results are reported as absolute values with 95% confidence intervals (95% CIs). Sensitivity and specificity were calculated using the following formulas: TP (true positive)/TP + FN (false negative) and TN (true negative)/TN + FP (false positive), respectively. The PPV represents the TP/TP + FP ratio, while the NPV was calculated as TN/TN + FN.

## 3 Results

### 3.1 General characteristics

One hundred fourteen patients with a suspected acute spinal infection agreed to undergo sampling and screening; ninety-two patients were diagnosed with a definite acute spinal infection. The duration of symptoms in the included patients was 11.58 ± 3.8 days (min: 3 days, max: 20 days). Fourteen were diagnosed with a clinical acute spinal infection, and 8 patients had a noninfectious disease, such as a tumor. In 32/114 (28.1%) patients, pathogenic microorganisms were identified by culture (blood or/and tissue). In 44/114 (38.6%) patients, pathogenic microorganisms were identified using conventional methods (CMT: culture and/or smear). Pathogenic microorganisms were detected in ninety patient tissue specimens using mNGS. Forty-six specimens were identified as positive only using mNGS.

No significant differences in baseline characteristics (Table 1) were observed between the definite acute spinal infection, clinical acute spinal infection, and noninfection groups. Eighty-two (71.93%) of the patients were treated with antibiotics prior to admission, and 34 (41.46%) of them changed antibiotics based on the mNGS results during hospitalization.

**Table 1:** General characters of different groups.

GROUP	Infection	Noninfection	Clinical infection	P value
Age (years)	62 ± 11.73	55.25 ± 9.54	55.07 ± 20.67	P > 0.05
Sex (males/total patients)	0.53%	0.50%	0.57%	
Sample Type (CT/OP)	52/40	2/6	10/4	
Temperature	38.74 ± 0.4	38.7 ± 0.23	38.70 ± 0.44	
WBC	9.71 ± 4.15	9.13 ± 3.23	10.69 ± 3.97	
DAYSATB	4.03 ± 2.9	3.5 ± 3.02	3 ± 2.72	
CRP	90.21 ± 81.64	37.16 ± 3.19	76.31 ± 61.91	
ESR	62.91 ± 35.81	53.5 ± 13.13	46.36 ± 31.44	

WBC: white blood cell count; DAYSATB: days of antibiotic use; CRP: C-reactive protein; ESR: erythrocyte sedimentation rate; CT: CT-guided percutaneous biopsy; OP: intraoperative tissue biopsy.

### 3.2 Diagnostic value of mNGS for acute spinal infection

Among the 114 patients, both mNGS and CMT were positive in 42 (36.8%) samples, and both were negative in 14 (12.3%) samples. Using CMT (culture and/or smear) as the gold standard, the sensitivity, specificity, PPV and NPV of mNGS are shown in Table 2.

**Table 2:** Diagnostic performance of mNGS compared with culture and smear for the detection of spinal infectious pathogens.

Conventional test methods	mNGS			
	Sensitivity (%)	Specificity (%)	PPV	NPV
Culture	93.8	26.8	33.3	91.7
Culture + Smear	95.5	31.4	46.7	91.7

Culture: refers to blood and/or tissue culture; Smear: refers to blood and/or tissue smear; PPV: positive predictive value; NPV: negative predictive value.

The positive percent agreement between the mNGS result and final diagnosis (84.91%, 95% CI: 76.34%–96.7%) was significantly higher than that between culture and final diagnosis (30.19%, 95% CI: 21.85%–39.99%) and CMT and final diagnosis (43.40%, 95% CI: 31.39%–49.97%) (P < 0.05). For the culture-positive and CMT-positive (without culture) groups, the consistency between mNGS and the culture groups was 93.75% and 100%, respectively. mNGS detected forty-six more pathogens than CMT, and the diagnostic rate increased by 43.40% (46/106). Overall, mNGS showed a sensitivity of 97.83% (90/92) (95% CI, 91.62%–99.62%) and a specificity of 100% (8/8) (95% CI, 59.77%–100%) when subjects with clinical and definite infections were defined as patients, and patients with tumors were defined as controls. The NPV and PPV were 80% (95% CI, 44.22%–96.46%) and 100% (95% CI, 94.89%–100%), respectively.

**Fig. 1:**
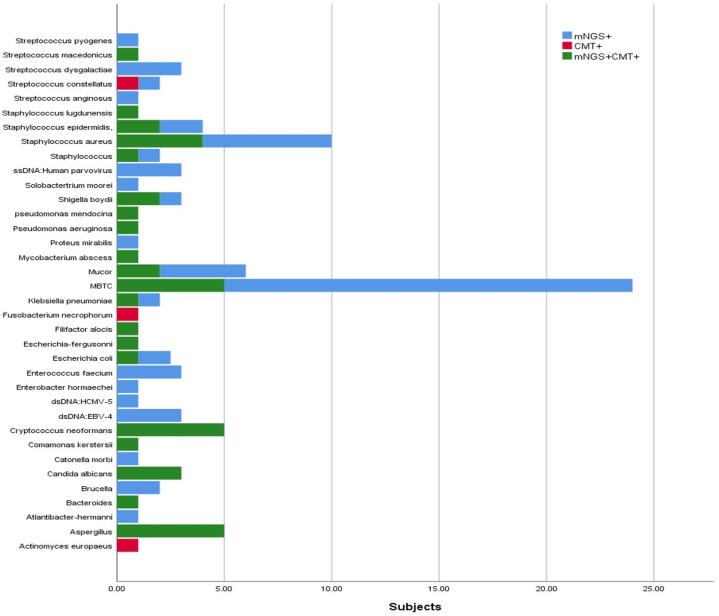
The consistency of mNGS with CMT.

The consistency of mNGS with CMT is shown in Fig. 1.

### 3.3 Effect of preadmission antibiotic use on the mNGS and CMT results

Eighty-two patients received antibiotics before admission; compared to the group that did not receive antibiotic treatment, a significant decrease in the positive percent agreement of mNGS was not observed (80.49% vs. 75%, P = 0.52). In comparison, culture positivity (20.73% vs. 46.88%, P < 0.05) showed a decreasing trend in the prior treated group. In our study, the duration of antibiotic use before admission had no significant effect on the positive rates of mNGS and culture.

### 3.4 Identification of pathogen species in culture-/smear-negative samples using mNGS

In the present study, sixty samples were negative using CMT, and associated patients were diagnosed with a clinical acute spinal infection based on only clinical symptoms. Among the sixty patients with a clinical acute spinal infection, 46 were positive for pathogens using mNGS, which indicated 26 cases of bacterial infection, 20 cases of MBTC infection, 3 cases of fungal infection, 8 cases of viral infection, and 9 cases of polymicrobial infection.

**Fig. 2:**
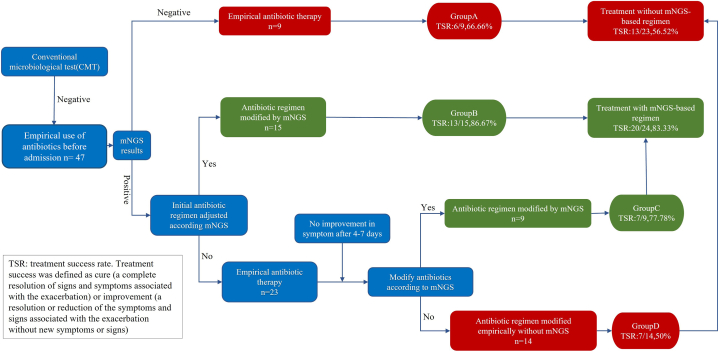
Diagnostic value of mNGS in CMT-negative patients. (A) Schematic diagram of the different classification processes, showing whether the diagnosis and treatment processes were guided by the mNGS results (classification processes A, B, C and D in the figure). Empiric antibiotic treatment was administered within 24 h of admission. All patients were closely monitored for reactions, adverse reactions, the emergence of secondary infections and improvements in symptoms once they started empiric antibiotic treatment. For patients with poor symptom improvement, we considered modifying the initial antibiotic regimen.

**Fig. 3:**
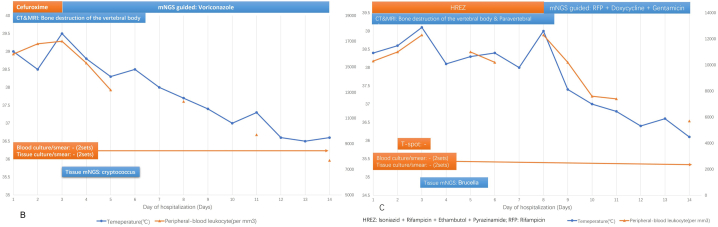
B: Representative case from group B. A 49-year-old female patient developed a fever and back pain for 6 days. CT-guided paravertebral abscess puncture was performed on the day of admission to obtain a tissue sample. Cefuroxime was given empirically before admission, but the symptoms did not improve after 4 days of treatment. The antibiotic treatment was changed to voriconazole on the third day of admission based on the mNGS test results (cryptococcus). After 8 days of therapy, the patient’s symptoms improved. C: Representative case from group C. A 71-year-old male patient developed high fever and low back pain. The patient lives in a pastoral area with a history of cattle and sheep contact. Anti-tuberculosis treatment was administered at the local hospital, and the original regimen (HREZ) was continued for 3 days after admission. On the third day of admission, mNGS results suggested Brucella infection, and the antibiotic regimen was not changed because Brucella is sensitive to rifampicin. After 5 days of poor results, the antibiotic regimen was changed to rifampicin + doxycycline + gentamicin, and the patient's symptoms improved after 3 days.

In the absence of pathogenic evidence, the use of targeted and precise antibiotic regimens remains a major challenge for patients with a suspected acute spinal infection. We reviewed the data from forty-seven patients who received antibiotic therapy before admission and their clinical results on day 14 to assess the potential benefits of mNGS in guiding antibiotic treatment regimens. The forty-seven patients were divided into 4 groups to determine whether the results of mNGS modified the antibiotic regimen; the respective diagnosis and treatment procedures are shown in Fig. 2. The treatment success rate (TSR) on the 14th day after admission was evaluated using standard procedures (Table 3), and the differences between the groups were compared (A, B, C, and D). Fig. 3 (B C) shows representative cases from groups B and C to visualize the role of mNGS in guiding the antibiotic regimen.

A. Nine patients with negative mNGS results were treated with empiric antibiotics; the total TSR in this group was 66.66% (6/9).
B. Fifteen patients received an adjustment of their initial empiric antibiotic regimen immediately after receiving mNGS-positive results; the TSR was 86.67% (13/15).
C. Nine patients were initially treated with empiric antibiotics, but when symptoms did not improve after 4–7 days of treatment, the initial treatment plan was modified according to the results of mNGS; the TSR in this group was 77.78% (7/9).
D. The antibiotic regimens of fourteen patients were not adjusted by referring to the mNGS results during the entire treatment duration. If symptoms did not improve 4–7 days after the initial antibiotic treatment, the antibiotic regimen was modified, but the mNGS results were not used as a reference. Antibiotic use was consistent with the mNGS results in 6 of the 14 patients. The final TSR was 50% (7/14) after 14 days.

**Table 3:** Evaluation of Treatment Success Rate (TSR).

**Participants**	Inclusion criteria: patients 18 years of age or older with a suspected acute spinal infection who underwent mNGS
	Exclusion criteria:
	1. Patients aged less than 18 years;
	2. Known pregnancy;
	3. Psychiatric disorders or inability to provide written informed consent, not available for follow-up; and
	4. Patients suffering from severe organ dysfunction.
	This analysis included 114 of 121 participants who completed follow-up.
**Outcomes**	Antibiotic use
	TSR on day 14 after admission: TSR was defined as cure (a complete resolution of signs and symptoms associated with the exacerbation) or improvement (a resolution or reduction of the symptoms and signs associated with the exacerbation without new symptoms or signs).
	1. Cure was defined as the resolution of clinical, laboratory, and radiographic signs of infection.
	2. Improvement was defined as reduction of clinical signs and symptoms, improvement of laboratory findings, and reduction in the number or intensity of radiographic signs of infection.
	3. Treatment success represented the sum of the rates for cure and improvement.
	4. Treatment failure included death, recurrence, relapse, or persistence of clinical, laboratory, and radiologic signs of acute spinal infection and participants who were lost to follow-up.

Overall, in the groups for whom antibiotic regimens were based on or modified according to the mNGS results (B + C), the overall TSR was 83.33% (20/24). This value was significantly higher than that in patients treated with antibiotics without reference to the mNGS results (56.52%, 13/23; A + D) (P = 0.04). Based on these results, mNGS may play vital roles in improving medical decision-making and optimizing antibiotic treatment, particularly in patients with an acute spinal infection for whom negative results were recorded using conventional tests.

### 3.5 Distributions of identified pathogens

**Fig. 4:**
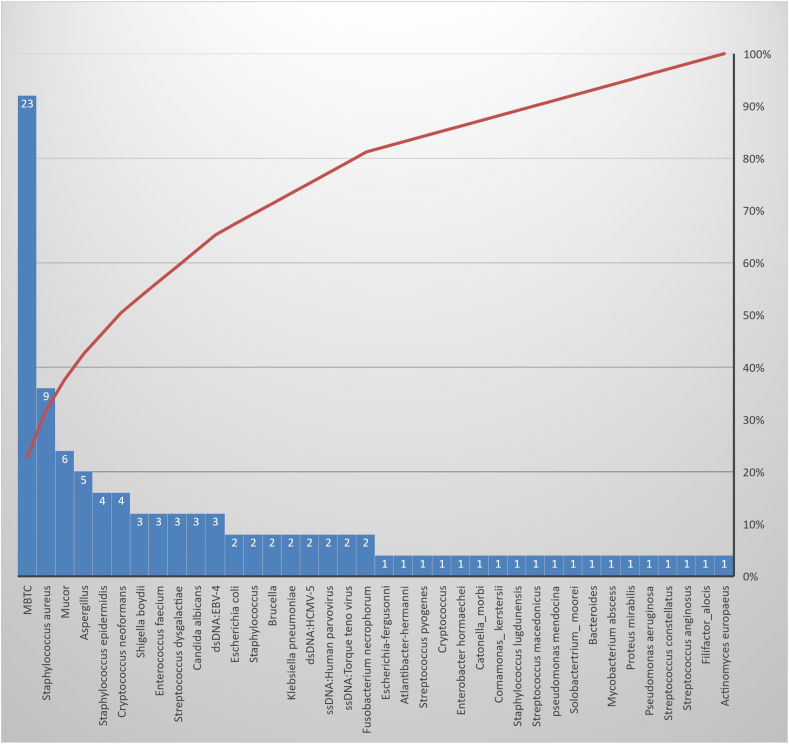
Distribution of identified pathogens.

*Mycobacterium tuberculosis* complex (MTBC) was the most frequently detected pathogen in this study, followed by *Staphylococcus aureus* and *Mucor* sp. (Fig. 4). mNGS was superior to culture in the identification of pathogens with a low culture rate or demanding and time-consuming culture processes, such as viruses, mycoplasma, and MTBC.

Background microorganisms were divided into two groups (Table 4). Some were microorganisms that commonly colonize the body surface or in the environment, such as *Propionibacterium acnes;* the other was microorganisms detected by mNGS, but no evidence for the infection or colonization of these microorganisms has been reported previously.

**Table 4:** Background microbial distribution.

Background pathogen	Count
*Propionibacterium acnes*	34
*Staphylococcus epidermidis*	16
*Staphylococcus hominis*	14
*Acinetobacter johnsonii*	12
*Brevundimonas vesicularis*	11
*Micrococcus luteus*	9
*Ralstonia insidiosa*	9
*Moraxella osloensis*	8
*Sphingomonas echinoides*	8
*Malassezia restricta*	8
*Cupriavidus metallidurans*	6
*Acidovorax*	6
*Burkholderia vietnamiensis*	6
*Propionibacterium humerusii*	5
*Brevundimonas*	5
*Ralstonia pickettii*	5
*Cladosporium sphaerospermum*	5
*Pseudomonas fluorescens*	5
*Cutibacterium granulosum*	4
*Staphylococcus haemolyticus*	4
*Acinetobacter lwoffii*	3
*Novosphingobium lentum*	3
*Burkholderia pyrrocinia*	2
*Porphyrobacter*	2
*Acinetobacter guillouiae*	2
*Corynebacterium aurimucosum*	2
*Campylobacter mucosalis*	2
*Cupriavidus pauculus*	2
*Sphingobium xenophagum*	2
*Staphylococcus warneri*	2
*Acinetobacter ursingii*	2
*Corynebacterium accolens*	2
*Aquabacterium parvum*	1
*Caballeronia sordidicola*	1
*Curvibacter lanceolatus*	1
*Curvibacter*	1
*Methylorubrum extorquens*	1
*Mycolicibacterium*	1
*Mycolicibacterium malmesburyense*	1
*Pelomonas puraquae*	1
*Cupriavidus basilensis*	1
*Chryseobacterium scophthalmum*	1
*Microbacterium laevaniformans*	1
*Acidovorax delafieldii*	1
*Methylobacterium brachiatum*	1
*Paracoccus salipaludis*	1
*Proteus hauseri*	1
*Cupriavidus gilardi*	1
*Chryseobacterium*	1
*Bradyrhizobium*	1
*Methylobacterium radiotolerans corrig.*	1
*Staphylococcus*	1
*Sphingobium*	1
*Cladosporium sphaerospermum*	1
*Malassezia globosa*	1
*Brevundimonas diminuta*	1
*Cupriavidus gilardi*	1
*Pseudomonas oleovorans*	1
*Staphylococcus capitis*	1
*Acidovorax temperans*	1
*Burkholderia ubonensis*	1
*Acinetobacter parvus*	1
*Paracoccus sanguinis*	1
*Xanthomonas campestris*	1
*Variovorax paradoxus*	1
*Gordonia bronchialis*	1

### 3.6 Detection time (mNGS vs. traditional culture/smear)

We compared the time required for pathogen identification between culture/smear and mNGS; the mNGS identification time was approximately 29–53 h. Positive gram-staining or smear results were regularly available within 24 h. However, the positivity rates were low, and these methods did not identify bacteria at the species level. Regarding the time for microbial identification, the mNGS method took an average of 40.67 h per sample, which has obvious advantages compared with the traditional culture method (90.88 h).

## 4 Discussions

This retrospective study assessed the value of mNGS in the detection of pathogens causing acute spinal infections. Rapid and accurate pathogen detection is essential for the clinical diagnosis and appropriate treatment of acute spinal infections. Research has shown that mNGS technology is valuable for the diagnosis and treatment of different infectious diseases [7,10,17]. Because of the importance of accurate identification of the causative agent in the diagnosis and treatment of acute spinal infections, the overall pros and cons of the role of mNGS in the treatment of acute spinal infections are worth exploring.

Compared with the combined culture and smear test, which is regarded as the gold standard, mNGS showed high sensitivity and a high NPV. According to previous studies, mNGS had different sensitivities and specificities in the identification of different types of pathogens. For bacteria, the sensitivity ranged from 50.7 to 100%, and the specificity ranged from 76.5 to 87.5% [7,11]. In the present study, mNGS did not show high specificity relative to high sensitivity, as in previous studies. We believe that the main explanation for this difference is the low sensitivity of culture and smear, which may lead to many false negative results and thus the low specificity of mNGS. In this study, we propose that the reasons for the false-negative results of culture and smear are as follows: 1. at the time when some specimens were collected, the patient had progressed past the acute phase and was in the stable phase; 2. some patients were finally confirmed to have infections caused by viruses, fungi or bacteria that are difficult to culture, such as acid-fast bacilli; and 3. some patients had been treated with antibiotics at the time of sampling. These factors may jointly lead to the low specificity and PPV of mNGS compared with CMT, the gold standard.

Considering the final clinical diagnosis as a reference, the positive percent agreement of mNGS (84.91%) was significantly higher than those of culture and CMT (excluding culture) (30.19%, 43.40%, P < 0.05). In 76.67% of the patients with a CMT-negative acute spinal infection, mNGS detected the presence of pathogenic microorganisms. In culture-positive samples, mNGS identified approximately 93.75% of the pathogens, and the results were similar to those reported in published articles [18,19]. One of the explanations for the high diagnostic value of mNGS may be the ability of mNGS to directly detect gene fragments of pathogens, whereas culture requires pathogens to survive and multiply. Another explanation is that some patients received effective treatment before this admission. Our research has found that treatment will lead to a decrease in the positivity rate of culture.

Spinal tuberculosis is a common extrapulmonary tuberculosis disease. Although it has a high long-term incidence, no clear guidelines for diagnosis and treatment are available. Early diagnosis and effective treatment are necessary to prevent permanent neurological dysfunction and reduce spinal deformity [20,21].

Although the positive detection of mycobacteria in culture is the gold standard for the diagnosis of spinal tuberculosis, the low detection rate and the 2- to 8-week period required for detection limit its role in early diagnosis [22]. In the present study, mNGS detected MBTC in 24 samples within 49 h, while only 14 of the corresponding samples were positive on CMT. The positive percent agreement of mNGS was significantly higher than that of CMT (100% vs. 58.33%, p < 0.01).

Two false-negative results were obtained in our study. The missed detection of *Actinomyces europaeus* and *Fusobacterium necrophorum* was due to the absence of corresponding sequences in our reference database. Thus, a variety of detection methods must be used and the database must be upgraded to prevent missed or misidentification and to improve the accuracy of pathogen detection.

A variety of antibiotics are currently used to treat acute spinal infections. Prior to pathogen isolation, broad-spectrum antibiotics or a combination of drugs, such as third-generation cephalosporins or fluoroquinolones plus clindamycin or vancomycin, should be empirically administered. Antibiotics should be subsequently adjusted according to the results of pathogen isolation [3]. However, some studies have observed that the isolation rate of traditional methods, such as culture, is low [[23], [24], [25]], which potentially hinders the optimization of antibiotic therapy. According to whether the antibiotic regimen referred to the results of mNGS, we compared different clinical strategies; the results showed that mNGS has the potential to optimize the use of antibiotics (B + C). Among infected patients for whom conventional laboratory evidence was lacking, the ability of mNGS to detect pathogens that CMT could not identify was emphasized; therefore, mNGS may help optimize medical decisions and antibiotic regimens. Although CMT is still the gold standard in many studies and mNGS has not yet achieved a similar clinical status, the diagnostic performance of mNGS shows that mNGS has essential value as a supplement to CMT, especially in patients for whom conventional tests remain negative.

Of the eight patients who were finally diagnosed with tumors, 5 patients experienced empirical antibiotic treatment. This antibiotic treatment was a waste of medical resources, and patients may have experienced unnecessary suffering caused by some side effects. Since our research shows that mNGS has a high NPV, negative mNGS results may be used to exclude fever as a symptom of infection. This mNGS exclusion strategy may reduce the overuse of antibiotics in noninfected patients and shorten the duration of antibiotic treatment.

As described in other studies [26,27], background/contaminating microbial genes were detected in most samples in our study. The most common bacteria were *Propionibacterium acnes*, followed by *Staphylococcus epidermidis* and *Staphylococcus hominis.* mNGS has high sensitivity; thus, microorganisms in the environment, on the skin, or in reagents may cause contamination [28]. Therefore, clinicians should carefully interpret mNGS results based on patients' clinical information.

We divided background pathogens into two categories: those that have never been reported to cause tissue infection and those that are widespread in the environment and may nonpathogenically colonize the human body. When suspicious background microorganisms are detected, clinicians should carefully review the patient's medical history, infection route, and possible source of infection to assess the possibility of background microorganisms as new pathogens and to avoid missing novel pathogens.

This research further compared the identification time associated with mNGS, Changzheng Hospital's critical value report, strain culture, and other identification methods. The time needed for mNGS identification is mainly composed of three parts: 1. the time required for specimen submission (this time varies according to distance); 2. the time required for laboratory pathogen detection (at least 24 h); and 3. the time required to analyze and interpret the data (3 h). Overall, the time required for mNGS detection was approximately 29–53 h, which was shorter than the time needed for pathogen identification in most laboratories. However, mNGS does not provide drug sensitivity results for pathogens.

For pathogenic bacteria with low nutritional requirements and a wide range of culture conditions (such as *Staphylococcus*), pathogens can be identified within 24 h after culture and provide feedback to clinicians. However, for pathogens with high nutritional requirements and strict culture conditions (such as MBTC), the time required is often longer, and false-negative results may occur. Some rapid detection techniques developed in recent years, such as multiplex PCR assays (i.e., GeneXpert, FilmArray) [29], allow the detection of pathogens within 1 day, but some rare pathogens may be missed. Therefore, mNGS has excellent potential for the rapid detection of the abovementioned pathogens in patients with an acute spinal infection.

Overall, mNGS has potential advantages under the circumstances described below. First, mNGS can accurately identify rare or even previously unknown pathogens causing acute spinal infections. Second, mNGS can provide faster detection results for pathogenic microorganisms with high culture requirements and slow growth. Third, mNGS may be an effective alternative for pathogen detection technologies with high speed but a limited detection spectrum, such as PCR assays. However, mNGS also has its disadvantages. First, a single test is expensive (approximately $500 per test) and not reimbursed by health insurance, which is unaffordable for some patients. Second, the pathogen database is limited and may miss pathogens that are not included in the database.

mNGS also has shortcomings, such as bias in pathogenic results, mNGS can be used to identify disease-causing microorganisms by enabling the comparison of nucleic acid sequences in the tested samples with sequences obtained from a database. Therefore, the accuracy of the database and the analysis of the data are key to ensuring the accuracy of the final results. The database used in the study is a combination of public databases (NCBI, FDA-ARGOS, PATRIC) and specific pathogen databases (parasite, virus, fungal microbial databases), and redundant, duplicate and erroneous data were filtered out. Additionally, a background microbial database was generated using statistical analysis of a large amount of data obtained from common human colonizing microorganisms, experimental environments, and clinical samples. The database was made as completely and accurately as possible and was continuously updated. In this study, we adopted specific “judgment thresholds” by referencing previous results to help us distinguish pathogenic microorganisms from background microorganisms while minimizing selection bias.

After obtaining the mNGS test results, two senior physicians (Yu Chen; Wen Yuan) evaluated the results, and those that were consistent with the clinical presentation, such as Mycobacterium tuberculosis and Staphylococcus aureus, were easier to confirm. However, for some microorganisms that are not commonly observed clinically, we usually use the 4 methods described in Method section above to make a judgment.

In addition to the above measures, we reevaluate the mNGS report, but to prevent missing rare microorganisms (especially in the case of multiple infections, e.g., Mycobacterium tuberculosis and ssDNA viruses, such as Torque teno virus 19) and to take the test results into consideration, we utilize more caution when classifying the pathogenic microorganisms observed in the report as background microorganisms.

Admittedly, even with the above approach, we have only minimized confirmation bias and have not been able to eliminate it completely, but we believe that this level of bias is acceptable.

This study has some other limitations. First, the limited mNGS reference database may have led to the missed detection of some rare pathogens. Second, since this study employed a single-center, retrospective, cross-sectional design, some bias may have been introduced when patients were included, which may have affected the clinical analysis. Third, this study only included patients with acute spinal infections and failed to include patients with subacute and chronic acute spinal infections; thus, the results are limited. Last but not least, due to various reasons, such as the level of economic development and medical insurance system, the application of mNGS was limited for patients who might serve as blank controls, resulting in a small number of controls in this study. As the number of study subjects continues to increase, these limitations will be further addressed in future studies.

## 5 Conclusions

We retrospectively analyzed the data from 114 patients with an acute spinal infection and evaluated the diagnostic ability of mNGS; mNGS provides reliable results when an acute spinal infection is suspected. Although CMT is the gold standard, mNGS has high sensitivity and a high NPV. The positive percent agreement between the mNGS results and the clinical diagnosis was significantly higher than that between the conventional methods and the clinical diagnosis, and more pathogenic microorganisms were identified by mNGS. Antibiotic treatment before specimen collection may reduce the positivity rate of culture, while mNGS does not seem to be affected by antibiotics. For pathogens with a slow proliferation time or strict culture conditions, mNGS has diagnostic advantages, and the time required for mNGS identification is shorter than that for traditional culture and strain identification. When formulating and optimizing antibiotic treatment regimens for patients with an acute spinal infection, mNGS may provide valuable information.

### Author contribution statement

Wen Yuan: Conceived and designed the experiments.

Chen Wang; Yifei Gu: Performed the experiments; Analyzed and interpreted the data; Contributed reagents, materials, analysis tools or data; Wrote the paper.

Jinquan Hu: Performed the experiments; Analyzed and interpreted the data; Contributed reagents, materials, analysis tools or data.

Xinwei Wang: Performed the experiments.

Yu Chen: Conceived and designed the experiments; Performed the experiments.

## Funding statement

His research was supported by grants from the National Natural Science Foundation of China (81902235).

### Data availability statement

Data will be made available on request.

## Declaration of interest's statement

The authors declare that they have no known competing financial interests or personal relationships that could have appeared to influence the work reported in this paper.
