# Supplementary material for: Application of next-generation metagenomic sequencing in the diagnosis and treatment of acute spinal infections
Source: Heliyon. 2023 Feb 22;9(3):e13951. doi: 10.1016/j.heliyon.2023.e13951 (PMC9984843; doi:10.1016/j.heliyon.2023.e13951)
Supplement: Multimedia component 1 [file mmc1.docx]

| Patient | Age | Sex | mNGS | Reads | Q30（%） | mNGSTC（h） | Culture | Smear | CTC（h） | ATB(days) | Pre-Antibiotic | Antibiotic changed | Diagnosis |
| --- | --- | --- | --- | --- | --- | --- | --- | --- | --- | --- | --- | --- | --- |
| 1 | 63.00 | male | Staphylococcus aureus | 647 | 78.14 | 39 | (-) | (-) | / | 6.00 | Cefazolin Sodium | Vancomycin | spinal infection |
| 2 | 39.00 | male | (-) | / | / | / | (-) | (-) | / | 7.00 | HREZ | 0.00 | clinical infection |
| 3 | 31.00 | female | MBTC | 27 | 70.63 | 37.00 | (-) | MBTC | 24.00 | 6.00 | HREZ | 0.00 | spinal tuberculosis |
| 4 | 70.00 | male | (-) | / | / | / | (-) | (-) | / | 0.00 | 0.00 | 0.00 | clinical infection |
| 5 | 26.00 | female | (-) | / | / | / | (-) | (-) | / | 7.00 | Ceftriaxone Sodium | 0.00 | clinical infection |
| 6 | 62.00 | female | Shigella boydii | 39 | 87.56 | 42.00 | Shigella boydii | (-) | 96.00 | 0.00 | 0.00 | 0.00 | spinal infection |
| 7 | 82.00 | male | MBTC | 28 | 70.65 | 45.00 | (-) | (-) | / | 7.00 | Ampicillin Sodium+SulbactamSodium | HREZ | spinal tuberculosis |
| 8 | 53.00 | female | Enterococcus faecium,dsDNA:HCMV-5 | 44;11 | 72.47 | 42.00 | (-) | (-) | / | 4.00 | HREZ | Cefoperazone Sodium+Sulbactam Sodium | spinal infection |
| 9 | 65.00 | female | Escherichia coli | 12 | 84.32 | 37.00 | Escherichia coli | (-) | 86.00 | 0.00 | 0.00 | 0.00 | spinal tuberculosis |
| 10 | 72.00 | male | (-) | / | / | / | (-) | (-) | / | 4.00 | Amoxicillin | none | non-infection |
| 11 | 77.00 | male | (-) | / | / | / | (-) | (-) | / | 0.00 | 0.00 | 0.00 | clinical infection |
| 12 | 45.00 | female | (-) | / | / | / | (-) | (-) | / | 6.00 | Amoxicillin | 0.00 | clinical infection |
| 13 | 53.00 | female | Comamonas kerstersii | 136 | 68.97 | 41.00 | Comamonas kerstersii | (-) | 102.00 | 0.00 | 0.00 | 0.00 | spinal infection |
| 14 | 58.00 | male | Candida albicans | 132 | 77.58 | 50.00 | (-) | Candida albicans | 24.00 | 4.00 | Vancomycin | Itraconazole | spinal tuberculosis |
| 15 | 73.00 | male | Streptococcus dysgalactiae | 274 | 79.57 | 29.00 | (-) | (-) | / | 0.00 | 0.00 | 0.00 | spinal infection |
| 16 | 52.00 | female | (-) | / | / | / | (-) | (-) | / | 0.00 | 0.00 | 0.00 | non-infection |
| 17 | 55.00 | male | MBTC | 135 | 66.34 | 34.00 | (-) | (-) | / | 0.00 | 0.00 | 0.00 | spinal infection |
| 18 | 52.00 | male | Staphylococcus aureus | 5298 | 78.09 | 36.00 | Staphylococcus aureus | (-) | 81.00 | 5.00 | Vancomycin | 0.00 | spinal tuberculosis |
| 19 | 61.00 | male | Staphylococcus aureus | 1425 | 76.46 | 39 | (-) | (-) | / | 8.00 | Amoxicillin | Vancomycin | spinal infection |
| 20 | 79.00 | female | Staphylococcus epidermidis | 65 | 69.37 | 33.00 | Staphylococcus epidermidis | (-) | 97.00 | 7.00 | HREZ | Ceftriaxone Sodium | spinal infection |
| 21 | 69.00 | female | MBTC | 2177 | 65.45 | 45.00 | (-) | (-) | / | 3.00 | Ceftriaxone Sodium | HREZ | spinal infection |
| 22 | 53.00 | male | MBTC | 52 | 80.14 | 35.00 | (-) | (-) | / | 3.00 | Cefazolin | HREZ | spinal tuberculosis |
| 23 | 72.00 | male | Streptococcus macedonicus | 366 | 88.67 | 47.00 | Streptococcus macedonicus | (-) | 104.00 | 0.00 | 0.00 | 0.00 | spinal infection |
| 24 | 67.00 | male | Candida albicans | 67 | 78.86 | 35.00 | Candida albicans | (-) | 96.00 | 5.00 | Ceftriaxone Sodium | Itraconazole | spinal infection |
| 25 | 48.00 | female | Aspergillus | 368 | 85.65 | 42.00 | (-) | Aspergillus | 24.00 | 3.00 | Ceftriaxone Sodium | Voriconazole | spinal infection |
| 26 | 44.00 | male | Shigella boydii;Escherichia coli | 58;16 | 72.31 | 47.00 | (-) | (-) | / | 4.00 | Cefradine | Levofloxacin | spinal infection |
| 27 | 58.00 | male | Mucor | 241 | 67.87 | 32.00 | (-) | (-) | / | 5.00 | Ampicillin Sodium+SulbactamSodium | Voriconazole | spinal infection |
| 28 | 44.00 | female | Staphylococcus | 430 | 75.45 | 29.00 | Staphylococcus | (-) | 110.00 | 4.00 | Ampicillin Sodium+SulbactamSodium | 0.00 | spinal tuberculosis |
| 29 | 67.00 | female | Streptococcus pyogenes;dsDNA:HCMV-5 | 323;36 | 75.47 | 44.00 | (-) | (-) | / | 5.00 | Ceftriaxone Sodium | Ampicillin Sodium+SulbactamSodium | spinal infection |
| 30 | 69.00 | male | Staphylococcus aureus;Mucor | 1254;360 | 87.33 | 41.00 | (-) | (-) | / | 9.00 | Ampicillin Sodium+SulbactamSodium | Ampicillin Sodium+SulbactamSodium+Amphotericin B | spinal infection |
| 31 | 65.00 | female | pseudomonas mendocina | 237 | 75.83 | 44.00 | pseudomonas mendocina | (-) | 88.00 | 0.00 | 0.00 | 0.00 | spinal infection |
| 32 | 74.00 | male | Staphylococcus aureus;Torque teno virus | 85;15 | 79.9 | 35.00 | (-) | (-) | / | 6.00 | HREZ | Vancomycin | spinal infection |
| 33 | 82.00 | male | (-) | / | / | / | (-) | (-) | / | 3.00 | HREZ | 0.00 | clinical infection |
| 34 | 71.00 | female | Enterococcus faecium | 254 | 70.20 | 42.00 | (-) | (-) | / | 5.00 | Cefoperazone Sodium+Sulbactam Sodium | 0.00 | spinal infection |
| 35 | 57.00 | female | Aspergillus | 400 | 76.62 | 41.00 | Aspergillus | (-) | 82.00 | 8.00 | HREZ | Voriconazole | spinal infection |
| 36 | 68.00 | male | Brucella,dsDNA:EBV-4 | 12;3 | 76.61 | 41.00 | (-) | (-) | / | 9.00 | Ceftriaxone Sodium | Doxycycline+Gentamicin+Rifampicin | spinal infection |
| 37 | 68.00 | male | MBTC | 61 | 63.25 | 42.00 | (-) | (-) | / | 6.00 | Cefradine | Ampicillin Sodium+SulbactamSodium | spinal tuberculosis |
| 38 | 71.00 | female | Escherichia-fergusonni | 74 | 88.17 | 44.00 | Escherichia-fergusonni | (-) | 91.00 | 0.00 | 0.00 | 0.00 | spinal infection |
| 39 | 62.00 | male | Staphylococcus epidermidis | 623 | 69.79 | 36.00 | (-) | (-) | / | 5.00 | Ceftriaxone Sodium | Ampicillin Sodium+SulbactamSodium | spinal infection |
| 40 | 41.00 | male | (-) | / | / | / | (-) | (-) | / | 5.00 | Ceftriaxone Sodium | none | non-infection |
| 41 | 31.00 | female | MBTC | 217 | 66.39 | 38.00 | (-) | MBTC | 24.00 | 7.00 | HREZ | 0.00 | spinal tuberculosis |
| 42 | 71.00 | female | (-) | / | / | / | (-) | (-) | / | 0.00 | 0.00 | 0.00 | clinical infection |
| 43 | 29.00 | male | (-) | / | / | / | (-) | (-) | / | 4.00 | HREZ | 0.00 | clinical infection |
| 44 | 54.00 | male | MBTC | 36 | 76.35 | 35.00 | (-) | (-) | / | 0.00 | 0.00 | 0.00 | spinal infection |
| 45 | 81.00 | male | MBTC | 30 | 78.69 | 44.00 | (-) | (-) | / | 7.00 | Vancomycin | HREZ | spinal tuberculosis |
| 46 | 51.00 | female | Catonella morbi,dsDNA:EBV-4 | 87;14 | 88.66 | 45.00 | (-) | (-) | / | 3.00 | Ceftriaxone Sodium | Levofloxacin | spinal infection |
| 47 | 65.00 | female | MBTC | 71 | 83.14 | 49.00 | (-) | (-) | / | 4.00 | Vancomycin | HREZ | spinal tuberculosis |
| 48 | 46.00 | female | (-) | / | / | / | (-) | (-) | / | 7.00 | Ceftriaxone Sodium | none | non-infection |
| 49 | 73.00 | female | (-) | / | / | / | (-) | (-) | / | 0.00 | 0.00 | 0.00 | clinical infection |
| 50 | 43.00 | male | (-) | / | / | / | (-) | (-) | / | 3.00 | HREZ | 0.00 | clinical infection |
| 51 | 56.00 | female | Pseudomonas aeruginosa | 410 | 83.47 | 49.00 | Pseudomonas aeruginosa | (-) | 93.00 | 0.00 | 0.00 | 0.00 | spinal infection |
| 52 | 55.00 | female | MBTC | 115 | 67.24 | 39.00 | (-) | (-) | / | 8.00 | Cefradine | HREZ | spinal tuberculosis |
| 53 | 73.00 | male | MBTC | 47 | 86.69 | 39 | (-) | (-) | / | 0.00 | 0.00 | 0.00 | spinal infection |
| 54 | 56.00 | male | (-) | / | / | / | (-) | (-) | / | 0.00 | 0.00 | 0.00 | non-infection |
| 55 | 57.00 | male | MBTC | 29 | 68.72 | 37.00 | (-) | (-) | / | 0.00 | 0.00 | 0.00 | spinal infection |
| 56 | 50.00 | male | MBTC | 58 | 66.48 | 48.00 | (-) | MBTC | / | 5.00 | HREZ | 0.00 | spinal tuberculosis |
| 57 | 63.00 | female | Staphylococcus aureus | 932 | 74.41 | 35.00 | (-) | (-) | / | 6.00 | Cefazolin Sodium | Vancomycin | spinal infection |
| 58 | 79.00 | male | Staphylococcus aureus | 2363 | 70.17 | 34.00 | Staphylococcus aureus | (-) | 99.00 | 8.00 | Cefradine | Vancomycin | spinal infection |
| 59 | 69.00 | female | MBTC | 64 | 79.32 | 41.00 | (-) | (-) | / | 3.00 | Cefazolin | Ampicillin Sodium+SulbactamSodium | spinal tuberculosis |
| 60 | 55.00 | male | MBTC | 64 | 76.83 | 41.00 | (-) | MBTC | / | 7.00 | HREZ | 0.00 | spinal tuberculosis |
| 61 | 72.00 | female | Staphylococcus lugdunensis | 64 | 87.2 | 46.00 | Staphylococcus lugdunensis | (-) | 103.00 | 0.00 | 0.00 | 0.00 | spinal infection |
| 62 | 67.00 | male | Cryptococcus neoformans | 268 | 80.75 | 39.00 | Cryptococcus neoformans | (-) | 93.00 | 5.00 | Ampicillin Sodium+SulbactamSodium | Amphotericin B+Voriconazole | spinal infection |
| 63 | 50.00 | female | Aspergillus | 287 | 76.87 | 40.00 | (-) | Aspergillus | 24.00 | 0.00 | 0.00 | 0.00 | spinal infection |
| 64 | 47.00 | male | Mucor | 302 | 87.64 | 48.00 | (-) | (-) | / | 6.00 | Cefoperazone Sodium+Sulbactam Sodium | Voriconazole | spinal infection |
| 65 | 51.00 | male | Streptococcus constellatus | 1254 | 64.9 | 31.00 | (-) | (-) | / | 8.00 | HREZ | Cefoxitin sodium | spinal infection |
| 66 | 42.00 | male | Aspergillus | 263 | 85.36 | 40.00 | (-) | Aspergillus | 24.00 | 6.00 | Voriconazole | 0.00 | spinal tuberculosis |
| 67 | 67.00 | female | Solobactertrium moorei | 211 | 75.47 | 47.00 | (-) | (-) | / | 5.00 | Ampicillin | Ceftriaxone Sodium | spinal infection |
| 68 | 69.00 | male | Staphylococcus aureus | 785 | 67.7 | 35.00 | Staphylococcus aureus | (-) | 85.00 | 5.00 | Vancomycin | 0.00 | spinal infection |
| 69 | 65.00 | female | Staphylococcus epidermidis | 220 | 83.2 | 36.00 | (-) | (-) | / | 0.00 | 0.00 | 0.00 | spinal infection |
| 70 | 76.00 | male | Mycobacterium abscess |  | 86.41 | 53.00 | Mycobacterium abscess | (-) | 77.00 | 0.00 | 0.00 | 0.00 | spinal infection |
| 71 | 84.00 | male | (-) | / | / | / | Fusobacterium necrophorum | (-) | 84.00 | 7.00 | Cefoperazone Sodium+Sulbactam Sodium | 0.00 | spinal infection |
| 72 | 71.00 | female | MBTC | 5 | 83.21 | 52.00 | (-) | MBTC | / | 3.00 | HREZ | 0.00 | spinal infection |
| 73 | 56.00 | male | Cryptococcus neoformans | 187 | 67.46 | 39.00 | Cryptococcus neoformans | (-) | 87.00 | 8.00 | Ampicillin Sodium+SulbactamSodium | Amphotericin B+Fluconazole | spinal infection |
| 74 | 65.00 | female | Staphylococcus aureus,ssDNA:Human parvovirus | 567;26 | 58.01 | 41.00 | (-) | (-) | / | 3.00 | HREZ | Vancomycin | spinal infection |
| 75 | 68.00 | female | MBTC,dsDNA:EBV-4 | 16;5 | 79.20 | 49.00 | (-) | (-) | / | 5.00 | Ampicillin Sodium+SulbactamSodium | HREZ | spinal tuberculosis |
| 76 | 72.00 | female | Brucella | 15 | 79.73 | 49.00 | Streptococcus constellatus | (-) | 86.00 | 0.00 | 0.00 | 0.00 | spinal infection |
| 77 | 63.00 | male | Escherichia coli | 150 | 77.12 | 38.00 | (-) | (-) | / | 9.00 | Levofloxacin | 0.00 | spinal infection |
| 78 | 61.00 | female | (-) | / | / | / | (-) | (-) | / | 6.00 | Amoxicillin | none | non-infection |
| 79 | 31.00 | male | Aspergillus | 425 | 76.54 | 39.00 | (-) | Aspergillus | 24.00 | 5.00 | Voriconazole | 0.00 | spinal tuberculosis |
| 80 | 72.00 | female | (-) | / | / | / | (-) | (-) | / | 6.00 | Vancomycin | 0.00 | clinical infection |
| 81 | 27.00 | male | (-) | / | / | / | (-) | (-) | / | 3.00 | HREZ | 0.00 | clinical infection |
| 82 | 46.00 | male | Streptococcus anginosus | 2365 | 66.65 | 51.00 | (-) | (-) | / | 0.00 | 0.00 | 0.00 | spinal infection |
| 83 | 80.00 | female | MBTC | 52 | 74.56 | 47.00 | (-) | (-) | / | 3.00 | Ceftriaxone Sodium | HREZ | spinal tuberculosis |
| 84 | 53.00 | male | Staphylococcus epidermidis, | 134 | 70.83 | 42.00 | Staphylococcus epidermidis | (-) | 84.00 | 5.00 | Cefradine | Ceftriaxone Sodium | spinal infection |
| 85 | 65.00 | male | MBTC,ssDNA:Human parvovirus | 34;7 | 88.31 | 45.00 | (-) | (-) | / | 0.00 | Cefazolin | Ceftriaxone Sodium | spinal tuberculosis |
| 86 | 54.00 | male | (-) | / | / | / | (-) | (-) | / | 0.00 | 0.00 | 0.00 | non-infection |
| 87 | 73.00 | male | (-) | / | / | / | (-) | (-) | / | 0.00 | 0.00 | 0.00 | clinical infection |
| 88 | 44.00 | female | (-) | / | / | / | (-) | (-) | / | 0.00 | Ceftriaxone Sodium | 0.00 | clinical infection |
| 89 | 57.00 | female | Filifactor alocis | 25 | 88.00 | 52.00 | Filifactor alocis | (-) | 101.00 | 0.00 | 0.00 | 0.00 | spinal infection |
| 90 | 59.00 | male | Cryptococcus neoformans | 328 | 86.71 | 38.00 | (-) | Cryptococcus neoformans | 24.00 | 6.00 | Cefradine | Amphotericin B | spinal tuberculosis |
| 91 | 73.00 | male | Streptococcus dysgalactiae | 352 | 78.46 | 36.00 | (-) | (-) | / | 0.00 | 0.00 | 0.00 | spinal infection |
| 92 | 60.00 | female | (-) | / | / | / | (-) | (-) | / | 6.00 | Amoxicillin | none | non-infection |
| 93 | 56.00 | female | Atlantibacter-hermanni | 65 | 87.51 | 44.00 | (-) | (-) | / | 7.00 | HREZ | Levofloxacin | spinal infection |
| 94 | 51.00 | female | MBTC | 39 | 71.21 | 36.00 | (-) | (-) | / | 5.00 | Ceftriaxone Sodium | HREZ | spinal tuberculosis |
| 95 | 64.00 | female | Klebsiella pneumoniae | 53 | 84.36 | 37.00 | (-) | (-) | / | 4.00 | Cefoperazone Sodium+Sulbactam Sodium | 0.00 | spinal infection |
| 96 | 79.00 | male | Klebsiella pneumoniae | 10 | 71.87 | 37.00 | Klebsiella pneumoniae | (-) | 98.00 | 7.00 | HREZ | Cefoperazone Sodium+Sulbactam Sodium | spinal infection |
| 97 | 69.00 | male | Cryptococcus neoformans | 247 | 72.91 | 36.00 | Cryptococcus neoformans | (-) | 94.00 | 4.00 | Cefoperazone Sodium+Sulbactam Sodium | Amphotericin B+Fluconazole | spinal infection |
| 98 | 54.00 | female | MBTC | 18 | 73.65 | 40.00 | (-) | (-) | / | 6.00 | Ceftriaxone Sodium | HREZ | spinal tuberculosis |
| 99 | 78.00 | female | Enterobacter hormaechei | 74 | 72.33 | 45.00 | Enterobacter hormaechei | (-) | 96.00 | 0.00 | 0.00 | 0.00 | spinal infection |
| 100 | 67.00 | male | Candida albicans | 92 | 85.74 | 36.00 | Candida albicans | (-) | 86.00 | 7.00 | Levofloxacin | Itraconazole | spinal infection |
| 101 | 43.00 | male | Mucor | 97 | 78.66 | 29.00 | Mucor | (-) | 73.00 | 7.00 | Fluconazole | 0.00 | spinal infection |
| 102 | 49.00 | female | MBTC | 24 | 78.65 | 43.00 | (-) | (-) | / | 6.00 | Cefoxitin sodium | HREZ | spinal infection |
| 103 | 58.00 | male | Mucor,ssDNA:Human parvovirus | 34;6 | 78.11 | 42.00 | (-) | (-) | / | 7.00 | Ampicillin Sodium+SulbactamSodium | Voriconazole | spinal infection |
| 104 | 44.00 | male | MBTC | 57 | 68.34 | 43.00 | (-) | (-) | / | 5.00 | Cefradine | Ampicillin Sodium+SulbactamSodium | spinal tuberculosis |
| 105 | 67.00 | female | Proteus mirabilis | 182 | 89.21 | 48.00 | (-) | (-) | / | 6.00 | Cefradine | Ampicillin Sodium+SulbactamSodium | spinal infection |
| 106 | 69.00 | male | Cryptococcus neoformans | 88 | 73.85 | 39.00 | (-) | Cryptococcus neoformans | 24.00 | 5.00 | Cefradine | Amphotericin B | spinal infection |
| 107 | 65.00 | female | Staphylococcus aureus | 1325 | 70.97 | 43.00 | Staphylococcus aureus | (-) | 87.00 | 0.00 | 0.00 | 0.00 | spinal infection |
| 108 | 75.00 | male | Streptococcus dysgalactiae | 243 | 81.04 | 36.00 | (-) | (-) | / | 4.00 | Amoxicillin | Ceftriaxone Sodium | spinal infection |
| 109 | 83.00 | male | (-) | / | / | / | Actinomyces europaeus | (-) | 86.00 | 5.00 | Cefoperazone Sodium+Sulbactam Sodium | 0.00 | spinal infection |
| 110 | 71.00 | female | Staphylococcus | 1884 | 62.65 | 35.00 | (-) | (-) | / | 6.00 | Ampicillin | Ceftriaxone Sodium | spinal infection |
| 111 | 54.00 | male | Mucor | 506 | 76.01 | 35.00 | Mucor | (-) | 86.00 | 6.00 | Ceftriaxone Sodium | Amphotericin B | spinal infection |
| 112 | 66.00 | female | Enterococcus faecium | 146 | 72.68 | 40.00 | (-) | (-) | / | 3.00 | HREZ | Cefoperazone Sodium+Sulbactam Sodium | spinal infection |
| 113 | 68.00 | female | Shigella boydii | 76 | 74.85 | 35.00 | Shigella boydii | (-) | 90.00 | 0.00 | 0.00 | 0.00 | spinal tuberculosis |
| 114 | 72.00 | female | Bacteroides | 83 | 69.71 | 48.00 | Bacteroides | (-) | 87.00 | 0.00 | 0.00 | 0.00 | spinal infection |
| Q30: how many percent of the bases in this sequencing have a sequencing quality greater than 99.99%；mNGSTC: mNGS detection time consumption; CTC: CMT detection time consumption; ATB: Days of antibiotic use before admission | | | | | | | | | | | | | |
