# Supplementary material for: Application of next-generation metagenomic sequencing in the diagnosis and treatment of acute spinal infections
Source: Heliyon. 2023 Feb 22;9(3):e13951. doi: 10.1016/j.heliyon.2023.e13951 (PMC9984843; doi:10.1016/j.heliyon.2023.e13951)
Supplement: Multimedia component 2 [file mmc2.docx]

**1 Nucleic acid extraction**

The 500ul sample was transferred to a grinding tube pre-filled with glass beads, tightened the tube cap, fixed on the adapter of the grinder (Shanghai Jingxin Industrial Development CO., LTD, Shanghai, China), and run at 60HZ for 5 min. The ground supernatant was used for DNA/RNA extraction and purification (ZymoBIOMICS DNA/RNA Miniprep Kit, R2002), and the extraction method was performed according to the instructions.

**2 Enrichment of pathogen-specific nucleic acid fragments**

The extracted nucleic acid was enriched by multiplex pathogen qPCR detection kit (Shanghai Bingyuan Medical Technology Co., Ltd., SJ0101).The reagent of FJ contains pathogenic microorganism-specific capture primers, DNA polymerase, dNTP and salt ions. The enrichment reaction system (35ul) consists of 28ul FJ reagent and 7ul nucleic acid templates. After the reaction system is prepared, the following procedures are run on the PCR machine: (1) 55 °C, 15 min; （2） 95℃，30sec； (3) Perform 20 cycles: 95°C, 10sec; 63℃，1min； （4）72℃，5min； (5) 4 °C heat preservation.

**3 Multiplex qPCR reaction**

The A1-H3 in the kit are 24 qPCR reagents, each of which can be used to detect 4 targets, each labeled with FAM, VIC, Texas red, and Cy5 fluorophores, respectively. The A1-H3 qPCR reagents were pipetted 9ul into the loading wells of the PCR plate in a certain order, and then 1ul of the enrichment product of step 2 was added sequentially to each well using a continuous dispenser. After sealing the reaction system using a sealing film and instantaneously, run the following procedures on the Real-time PCR instrument: (1) 95°C, 5min; (2) Perform 35 cycles: 95°C, 10sec; 60 °C, 30 sec, and acquire fluorescence in the 60 °C renatured extension phase.

**4 Analysis of results**

if the CT value ≤ 25, it is a positive signal; (2) If the 25< CT value ≤ 32, it is a weak positive signal; (3) If the 32<CT value ≤ 35, it is a gray area signal, and this part of the signal is greatly disturbed by background noise; (4) If the curve does not peak, and the result is interpreted as NoCT, it is negative.
